# Stratification and prediction of remission in first-episode psychosis patients: the OPTiMiSE cohort study

**DOI:** 10.1038/s41398-018-0366-5

**Published:** 2019-01-17

**Authors:** Emanuela Martinuzzi, Susana Barbosa, Douglas Daoudlarian, Wafa Bel Haj Ali, Cyprien Gilet, Lionel Fillatre, Olfa Khalfallah, Réjane Troudet, Stéphane Jamain, Guillaume Fond, Iris Sommer, Stefan Leucht, Paola Dazzan, Philip McGuire, Celso Arango, Covadonga M. Diaz-Caneja, Wolfgang Fleischhacker, Dan Rujescu, Birte Glenthøj, Inge Winter, René Sylvain Kahn, Robert Yolken, Shon Lewis, Richard Drake, Laetitia Davidovic, Marion Leboyer, Nicolas Glaichenhaus, the OPTiMiSE Study Group

**Affiliations:** 1 Université Côte d’Azur, Centre National de la Recherche Scientifique, Institut de Pharmacologie Moléculaire et Cellulaire, Valbonne, France; 2 Université Paris Est Créteil, Faculté de Medicine Institut, National de la Santé et de la Recherche Médicale, Créteil, France; 3 Université Côte d’Azur, Centre National de la Recherche Scientifique, Laboratoire Informatique Signaux et Systèmes de Sophia Antipolis, Sophia Antipolis, France; 4 Assistance Publique Hôpitaux de Marseille, Marseille, France; 5 Department of Neuroscience and Department of Psychiatry, University Medical Center Groningen, Rijks Universiteit Groningen, Groningen, The Netherlands; 6 Department of Medical and Biological Psychology, University of Bergen, Bergen, Norway; 7 Department of Psychiatry and Psychotherapy, Technische Universität München, München, Germany; 8 Department of Psychosis Studies, Institute of Psychiatry, National Institute for Health Research, Mental Health Biomedical Research Centre, King’s College London, London, UK; 9 Child and Adolescent Psychiatry Department, Hospital General Universitario Gregorio Marañón, Universidad Complutense, Madrid, Spain; 10 Department of Psychiatry, Psychotherapy and Psychosomatic Medicine, Medical University Innsbruck, Innsbruck, Austria; 11 Department of Psychiatry, University of Halle, Halle, Germany; 12 Faculty of Health and Medical Sciences, Center for Neuropsychiatric Schizophrenia Research and Center for Clinical Intervention and Neuropsychiatric Schizophrenia Research, Psychiatric Hospital Center Glostrup, University of Copenhagen, Copenhagen, Denmark; 13 Department of Psychiatry, Brain Center Rudolf Magnus, UMC Utrecht, Utrecht, The Netherlands; 14 John Hopkins School of Medicine, The John Hopkins Hospital, Baltimore, USA; 15 Division of Psychology and Mental Health, School of Health Sciences, Faculty of Biology, Medicine and Health, Manchester Academic. Health Sciences Centre (MAHSC), University of Manchester, Manchester, UK; 16 Assistance Publique Hôpitaux de Paris, Pole de Psychiatrie et Addictologie, Hopitaux Universitaires Henri Mondor, Créteil, France; 17 Fondation Fondamental, Hôpital Albert Chenevier Pôle de Psychiatrie, Créteil, France

**Keywords:** Biomarkers, Physiology

## Abstract

Early response to first-line antipsychotic treatments is strongly associated with positive long-term symptomatic and functional outcome in psychosis. Unfortunately, attempts to identify reliable predictors of treatment response in first-episode psychosis (FEP) patients have not yet been successful. One reason for this could be that FEP patients are highly heterogeneous in terms of symptom expression and underlying disease biological mechanisms, thereby impeding the identification of one-size-fits-all predictors of treatment response. We have used a clustering approach to stratify 325 FEP patients into four clinical subtypes, termed C1A, C1B, C2A and C2B, based on their symptoms assessed using the Positive and Negative Syndrome Scale (PANSS) scale. Compared to C1B, C2A and C2B patients, those from the C1A subtype exhibited the most severe symptoms and were the most at risk of being non-remitters when treated with the second-generation antipsychotic drug amisulpride. Before treatment, C1A patients exhibited higher serum levels of several pro-inflammatory cytokines and inflammation-associated biomarkers therefore validating our stratification approach on external biological measures. Most importantly, in the C1A subtype, but not others, lower serum levels of interleukin (IL)-15, higher serum levels of C-X-C motif chemokine 12 (CXCL12), previous exposure to cytomegalovirus (CMV), use of recreational drugs and being younger were all associated with higher odds of being non-remitters 4 weeks after treatment. The predictive value of this model was good (mean area under the curve (AUC) = 0.73 ± 0.10), and its specificity and sensitivity were 45 ± 0.09% and 83 ± 0.03%, respectively. Further validation and replication of these results in clinical trials would pave the way for the development of a blood-based assisted clinical decision support system in psychosis.

## Introduction

Psychotic symptomatology includes loss of contact with reality, thought disorder, delusions and hallucinations, unusual or bizarre behavior, impaired social interactions and difficulties to carry out daily activities^1^. While psychosis could be caused by recreational drug use, physical illness or brain trauma, it is often symptomatic of the onset of severe psychiatric disorders such as schizophrenia, schizoaffective disorder or bipolar disorder.

According to current guidelines, first-line treatments of psychosis involve the use of the minimum effective dose of second-generation antipsychotics whenever possible. Whatever criteria are used to assess response to treatment, responses are highly heterogeneous. While 25–30% of first-episode psychosis (FEP) patients fully respond, a majority respond partially or not at all, and are therefore switched to second-line treatments^2^. As early response to treatment is one of the main factors associated with improved long-term prognosis^3–5^, identifying predictors of treatment response in FEP patients is an important issue in the field^6^. Response to treatment could be assessed either using predefined cutoffs in the percentage of reduction of baseline scores on a psychopathology rating scale^7^, or by measuring the proportion of patients meeting remission criteria. According to the definition proposed by the Remission in Schizophrenia Working Group (RSWG), remission can be defined by an absolute threshold of severity of symptoms in three dimensions: reality distortion, disorganization and negative symptoms^8^. Using this consensus definition, it was found that global functioning in the year before admission, the total score of the Strauss Carpenter Prognostic Scale and the Positive and Negative Syndrome Scale (PANSS) negative sub-score at admission were all predictive of symptom remission in cohorts of schizophrenia inpatients^9,10^. Despite these latter studies, clinicians still lack reliable predictors of remission in FEP patients.

Several environmental risk factors for psychosis have been identified^11^ including autoimmune disorders^12^ and infection with *Toxoplasma gondii*^13^, cytomegalovirus (CMV)^14^ and herpes simplex virus (HSV) type 1^15^. Meta-analyses have shown that drug-naive FEP patients exhibit altered serum levels of various cytokines compared to healthy individuals^16–18^. Since these data suggested a possible link between immune dysregulation and psychosis, it was proposed that serum levels of cytokines, chemokines and biomarkers of inflammation could predict early response to treatment^6^. However, antipsychotic treatments could impact cytokine levels including those of interleukin (IL)-1β, interferon (IFN)-γ, IL-12 and tumor necrosis factor (TNF)-α^19–23^. Therefore, the predictive value of serum biomarkers should be ideally assessed in minimally treated or untreated FEP patients, which is a challenge because of the difficulty to enroll these patients in clinical trials. This may be the reason for which only a few studies have investigated the association between baseline levels of peripheral biomarkers and remission in FEP patients. To identify biological predictors of remission in FEP patients, we have analyzed clinical data and biological samples from the multinational, multi-centered, randomized, double-blind “Optimization of Treatment and Management of Schizophrenia in Europe (OPTiMiSE)” study in which FEP patients were clinically assessed before and after 4 weeks of treatment with the second-generation antipsychotic amisulpride^24^. Our results demonstrate that serum levels of immune-related proteins before treatment combined with a few clinical variables could predict remission in at least a subtype of FEP patients.

## Materials and methods

### Patients

The OPTiMiSE study was conducted in 27 general hospitals and clinics in 14 European countries, Israel and Australia (Clinicaltrials.gov identifier is NCT01248195). FEP patients based on the EUFEST (European First Episode Schizophrenia Trial) study definition^25^ were recruited between May 2011 and April 2016 at the participating centers from nearby healthcare facilities. Eligible patients were aged 18–40 years and met criteria of the Diagnostic and Statistical Manual of Mental Disorders (4th edition) for schizophrenia, schizophreniform disorder or schizoaffective disorder. A total of 479 patients signed informed consent. Diagnoses were confirmed by the Mini International Neuropsychiatric Interview plus. Patients were excluded if more than 2 years had passed since the start of the FEP; if any antipsychotic drug had been used for more than 2 weeks in the previous year and/or for a total of 6-week lifetime; if patients had a known intolerance to one of the study drugs; if patients met any of the contraindications for any of the study drugs; if patients were coercively treated and/or represented by a legal guardian or under legal custody; or if patients were pregnant or breast feeding. Patients were required to provide written informed consent.

### Patient clinical assessment and primary outcome

A screening visit was conducted during which eligibility was assessed. Baseline data were obtained regarding demographics, diagnoses, current treatments and psychopathology: PANSS total score and sub-scores, overall severity of symptoms assessed using the Clinical Global Impression (CGI) scale^26^, depression assessed using the Calgary Depression Scale for Schizophrenia (CDSS)^27^ and social functioning assessed using the Personal and Social Performance Scale (PSP)^28^. Recreational drug use was also assessed. Data were collected at baseline and 4–5 weeks later.

All patients were treated for 4 weeks with up to 800 mg/day amisulpride in an open design. The primary outcome was symptomatic remission according to the criteria of Andreasen et al.^8^: a score of ≤3 (on a scale ranging from 1 to 7) simultaneously on 8 PANSS items: P1, P2, P3, N1, N4, N6, G5 and G9.

### Blood samples

Peripheral blood samples were obtained from fasting subjects between 7:00 am and 9:00 am. Five milliliters of peripheral blood were drawn by venipuncture into serum Vacutainer tubes. For the serum collection, the blood was allowed to clot for 1 h before centrifugation (1500 × *g*, 10 min). The serum and plasma samples were stored in 0.5 ml aliquots at −80 °C. For measuring protein and antibody levels, serum samples were thawed on ice, and 50 µl aliquots were prepared and stored at −80 °C.

### Immunoassay

Serum levels of IL-1α, IL-1β, IL-2, IL-4, IL-5, IL-6, IL-7, IL-8, IL-10, IL-12p40, IL- 12p70, IL-13, IL-15, IL-16 IL-17, IL-18, IL-21, IL-23, IL-27, IFN-γ, chemokines (C-C motif chemokine ligand (CCL)-2, CCL3, CCL4, CCL11, CCL13, CCL17, CCL19, CCL20, CCL22, CCL26, CCL27, and C-X3-C motif chemokine ligand (CX3CL)-1, CXCL10, CXCL11, CXCL12), TNF-α, TNF-β, granulocyte macrophage-colony stimulating factor, vascular endothelial growth factor (VEGF), C reactive protein (CRP), serum amyloid A protein (SAA), soluble intercellular adhesion molecule 1 (sICAM-1) and soluble vascular adhesion molecule 1 (sVCAM-1) were measured using the Pro-inflammatory Panel 1, Cytokine Panel 1, Chemokine Panel 1, Th17 Panel 1 and Vascular Injury Panel 2 v-PLEX® kits (MSD). All assays were performed according to the manufacturer’s instructions. The data were acquired on the V-PLEX® Sector Imager 2400 plate reader and analyzed using the Discovery Workbench 3.0 software (MSD). The standard curves for each cytokine were generated using the premixed lyophilized standards provided in the kits. Serial twofold dilutions of the standards were run to generate a 13-standard concentration set, and the diluent alone was used as a blank. The cytokine concentrations were determined from the standard curve using a 4-parameter logistic curve fit to transform the mean light intensities into concentrations. The lower limit of detection (LLOD) was determined for each cytokine and for each plate as the signal recorded for the blank plus 2 standard deviations (SDs).

### Serology

We measured plasma immunoglobulin G (IgG) antibodies reacting to HSV type 1, CMV and *T. gondii* using previously described immunoassay methods^29^. Diluted plasma was applied to antigens immobilized on the wells of microtiter plates and bound antibodies were quantified by means of reaction with enzyme-labeled anti-human IgG and the corresponding substrate. Reagents and assay kits for anti-HSV-1 were obtained from Focus Laboratories (USA). Anti-CMV and anti-*Toxoplasma* antibodies were obtained from IBL Laboratories (Germany). Results were obtained as quantitative values determined by comparison of the level of reactivity to standards run with each assay, as well as qualitative results listed as “positive” or “negative”.

### Statistical analysis

In univariate analysis, Mann–Whitney–Wilcoxon tests were performed to assess statistical significance of non-Gaussian distributed data. To develop a predictive model for remission we used the elastic net, which is a regularized regression model, i.e., general linear model with penalties to avoid extreme parameters that could cause overfitting^30^. Elastic net is also a method of selection of variables that addresses the issue of multicollinearity that arises in our dataset because cytokines and chemokines are not independent of each other. To minimize variation across testing datasets, we repeated fivefold cross-validation 100 times with independent random dataset partitions to optimize stability^31^. We tuned the hyper-parameters *α* and *λ* 10 times for each partition via fivefold cross-validation with the optimal tuning parameter values chosen to maximize the area under the receiver operating characteristics (ROC) curve (AUC)^32^. Weighted odds ratios (ORs) were calculated using the proportion of drawings in which the variable was selected as a weight. All statistical analyses were performed using the R software packages Stats^33^, Caret^34^, Glmnet^35^, pROC^36^ and eNetXplorer^37^_._

### Unsupervised statistical classification

To stratify *m* patients into *k* clusters based on their PANSS scores (*d* items per patient), we prepared a matrix X with one patient per line and one PANSS item per column (Supplementary Figure 1). Our objective was to find a matrix Y of labels. We thus tried to solve an optimization problem for finding a space which discriminated clusters based on a limited number of weighted PANSS items. The output was a W (weight) matrix with *k* columns and *d* lines computing the weight of each PANSS item. We achieved this goal using an alternating minimization procedure on Y and W in which we tried to minimize the Frobenius norm^38^.

## Results

### Soluble serum biomarkers did not predict remission in non-stratified FEP patients

A total of 479 patients were included in the OPTiMiSE clinical trial. Out of the 446 patients in the intention-to-treat sample, 371 completed amisulpride treatment. Among those, 325 had serum samples collected before the study treatment was initiated and were included in the present study (Table 1). Clinical assessment 3-4 weeks after treatment initiation revealed that 68.6% of the patients were in symptomatic remission^39^ according to the consensus definition^8^. As a first attempt to identify biomarkers that could predict remission, we analyzed serum samples for 43 interleukins, chemokines and biomarkers of inflammation. Among these proteins, 8 were below the LLOD in more than 10% of the samples and were not included in downstream analyses (Supplementary Table 1). In an exploratory analysis, we compared the levels of the 35 remaining proteins in remitters and non-remitters using univariate analysis. After correction for multiple test, none of these 35 proteins was present at different levels in remitters and non-remitters (Supplementary Table 2).

**Table 1:** Patient clinical characteristics

	Study sample as a whole			Patient subsets			
	All	Non-remitters	Remitters	C1A	C1B	C2A	C2B
Number of patients	325	102	223	97	62	95	71
Male (%)	70.2%	73.5%	68.6%	63.9%	83.9%	63.4%	75.3%
Age (years)	26.2 ± 6.2	25.0 ± 5.6	28.8 ± 6.4	25.9 ± 5.9	25.6 ± 5.6	27.0 ± 7.0	26.2 ± 6.0
BMI (kg/m^2^)	23.3 ± 4.2	23.5 ± 4.5	23.2 ± 4.1	23.3 ± 4.0	23.8 ± 3.8	23.2 ± 4.5	23.0 ± 4.5
Waist circumference (cm)	83.2 ± 11.8	84.6 ± 11.6	82.6 ± 11.9	83.2 ± 11.8	85.2 ± 11.6	82.3 ± 12.2	82.8 ± 10.3
PPANSS	19.8 ± 5.7	21.4 ± 5.4	19.1 ± 7.7	24.6 ± 4.1	17.2 ± 4.3	21.3 ± 4.1	14.0 ± 3.3
NPANSS	19.6 ± 7.1	22.5 ± 7.2	18.3 ± 6.7	25.7 ± 5.0	24.9 ± 3.9	14.5 ± 4.0	13.5 ± 4.0
GPANSS	38.2 ± 9.8	40.6 ± 8.8	37.0 ± 10.0	47.8 ± 6.8	37.5 ± 7.0	36.9 ± 6.3	27.6 ± 5.9
PANSS total	77.6 ± 19.0	84.5 ± 16.7	74.4 ± 19.6	98.1 ± 12.6	79.7 ± 9.8	72.6 ± 9.8	55.0 ± 9.6
CDSS	13.2 ± 4.8	13.2 ± 5.0	13.2 ± 4.7	14.1 ± 5.5	13.0 ± 4.8	13.7 ± 4.7	11.6 ± 3.3
PSP	48.9 ± 15.2	45.5 ± 15.6	50.5 ± 14.8	45.0 ± 13.2	44.9 ± 15.3	47.6 ± 14.1	59.2 ± 14.4
Recreational drug use	48.9%	48.0%	49.3%	39.2%	38.7%	53.8%	64.4%
Seropositivity to *T. gondii*	23.7%	30.2%	20.8%	30.9%	23.7%	17.4%	22.4%
Seropositivity to CMV	56.1%	56.3%	56.0%	60.6%	45.8%	60.9%	52.2%
Seropositivity to HSV-1	58.0%	56.3%	58.8%	63.8%	44.1%	64.1%	53.7%
Remitters	68.60%	0.00%	100.00%	57.70%	54.80%	61.30%	90.40%

The total number of patients, proportion of males, age (mean ± SEM), BMI (mean ± SEM), waist circumference (mean ± SEM) and clinical scores (mean ± SEM) before treatment are indicated in the study sample as a whole (all patients, non-remitters and remitters), and in the indicated subtypes. The proportion (%) of patients reporting recreational drug use, test seropositive for the indicated pathogens, or identified as remitters after 4 weeks of treatment with amisulpride are also indicated

*BMI* bone marrow index, *PANSS* Positive and Negative Syndrome Scale, *PPANSS* positive PANSS, *NPANSS* negative PANSS, *GPANSS* general psychopathology PANSS, *CDSS* Calgary Depression Scale for Schizophrenia, *PSP* Personal and Social Performance Scale, *CMV* cytomegalovirus, *HSV-1* herpes simplex virus type 1

In contrast to univariate methods that assess the differential expression of proteins on a single feature level, multivariate classification methods such as regularized logistic regression allows for establishing a prediction model based on samples with known class outcomes, e.g., remission versus non-remission^40^. A set of clinical and biological variables with the best joint discriminatory ability to differentiate between classes could be identified, and the resulting prediction model could then be used to predict the class outcomes of new patient samples. As a second attempt to predict remission, we investigated the association between serum protein levels and remission using regularized logistic regression after adjustment for age, gender, body mass index (BMI), waist circumference, use of recreational drugs and seropositivity to *T. gondii*, CMV and HSV-1. Applying this method to the dataset did not allow for identifying proteins whose serum levels were associated with increased odds of being non-remitters (not shown).

One obvious explanation for this negative result could be that none of the studied serum proteins is relevant for discriminating remitters and non-remitters among FEP patients. Alternatively, the heterogeneity of psychotic disorders in terms of symptomatology and likely etiology and pathophysiology may impede the identification of underlying remission predictors in a general population of FEP patients. To overcome this issue, we sought to stratify FEP patients based on their individual symptomatology assessed using the PANSS instrument^41^.

### Patient clustering

**Fig. 1:**
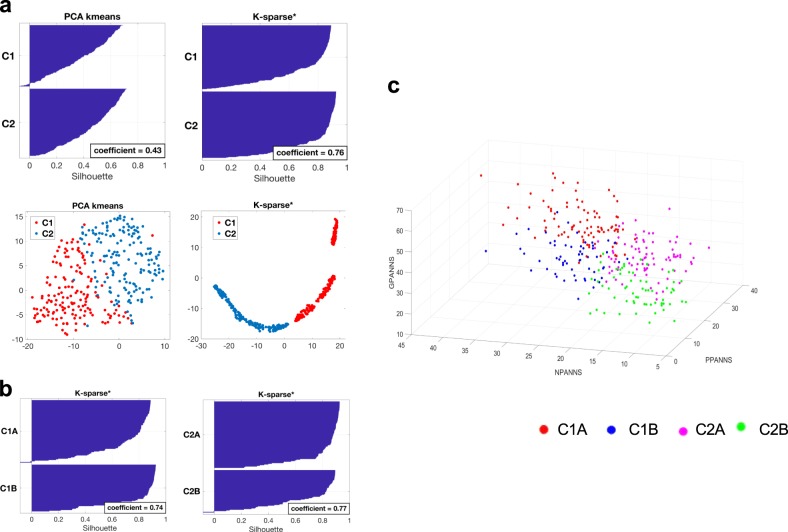
Clinical characteristics of patient subtypes. **a** First-level stratification. Silhouettes and *t*-distributed stochastic neighbor embedding (t-SNE) representations of patient clustering using principal component analysis (PCA)-*K-*means (left panels) and *K-*sparse* (right panels). Silhouette values could range from −1 to +1, with high values reflecting higher similarity within cluster. Mean silhouette values (coefficient) are indicated. **b** Second-level stratification. Silhouettes of C1 (left) and C2 (right) patient clustering using *K-*sparse*. Mean silhouette values (coefficient) are indicated. **c** Three-dimensional (3D) scatter plot representation of positive PANSS (PPANSS), negative PANSS (NPANSS) and general psychopathology PANSS (GPANSS) sub-scores of C1A, C1B, C2A and C2B patients. PANSS Positive and Negative Syndrome Scale

We sought to stratify patients in clusters in which patients within one cluster would be more similar (cohesion) than patients in the others (separation). We applied a two-step hierarchical unsupervised clustering method to a dataset consisting of the 30 individual PANSS scores of the 325 patients in the OPTiMiSE study sample, therefore resulting in four clusters: C1A and C1B, and C2A and C2B. We compared two methods for data clustering: principal component analysis (PCA)-*K-*means^42^ that is a popular method for cluster analysis, and *K-*sparse* that is a modified version of *K-*sparse^38^. While both methods were successful at stratifying the 325 patients of the OPTiMiSE study sample, *K-*sparse* outperformed PCA-*K-*means as demonstrated by both a higher mean silhouette value (0.76 compared to 0.43) and *t*-distributed stochastic neighbor embedding (t-SNE) graphical representations (Fig. 1a). We therefore selected *K-*sparse* for data clustering. First-level classification using *K-*sparse* identified two subtypes: C1 (*n* = 159) and C2 (*n* = 166). *K-*sparse* selected nine items that discriminated C1 and C2 patients, among which five belonged to the negative PANSS sub-scale (NPANSS) and four to the general psychopathology PANSS sub-scale (GPANSS) (Supplementary Table 3). Second-level classification identified four subtypes: C1A (*n* = 97) and C1B (*n* = 62) on one hand, and C2A (*n* = 95) and C2B (*n* = 71) on the other. *K-*sparse* selected eight PANSS items for discriminating C1A from C1B patients, among which four belonged to the positive PANSS (PPANSS) sub-scale and four to the GPANSS sub-scale (Supplementary Table 3). *K-*sparse* selected seven PANSS items for discriminating C2A from C2B patients, among which three belonged to the PPANSS sub-scale and four to the GPANSS sub-scale (Supplementary Table 3). In agreement with the nature and the weight of the PANSS items selected by *K-*sparse*, C1A and C1B patients exhibited more severe negative and general psychopathology symptoms compared to C2A and C2B patients respectively (Table 1). C1A and C2A patients exhibited more prominent positive and general psychopathology symptoms compared to C1B and C2B patients respectively (Table 1). Compared to other patients from the study sample, those from the C1A subtype exhibited more severe symptoms in the positive, negative and general psychopathology dimensions (Table 2, Fig. 1c). C1A patients also exhibited higher clinical scores as measured by the CGI and the CDSS and showed the worst psychosocial performance/functioning as measured by PSP scale (Table 2). In contrast, C2B patients exhibited less severe symptoms in the positive, negative and general psychopathology dimensions, exhibited lower CGI and CDSS scores and showed the best psychosocial performance/functioning as measured by the PSP scale (Table 2, Fig. 1c).

**Table 2:** Clinical score comparisons between individual patient subtypes

Clinical scores	C1A versus others			C1B versus others			C2A versus others			C2B versus others		
	C1A	Others	*P* value	C1B	Others	*P* value	C2A	Others	*P* value	C2B	Others	*P* value
PPANSS	24.56 ± 0.42	17.84 ± 0.33	<0.0001	17.21 ± 0.55	20.46 ± 0.36	<0.0001	21.29 ± 0.43	19.26 ± 0.40	0.002	13.97 ± 0.38	21.54 ± 0.32	<0.0001
NPANSS	25.72 ± 0.50	17.00 ± 0.42	<0.0001	24.92 ± 0.49	18.35 ± 0.44	<0.0001	14.48 ± 0.42	21.66 ± 0.47	<0.0001	13.49 ± 0.47	21.38 ± 0.43	<0.0001
GPANSS	47.77 ± 0.69	34.06 ± 0.51	<0.0001	37.52 ± 0.89	38.3 ± 0.64	0.52	36.86 ± 0.65	38.67 ± 0.71	0.14	27.55 ± 0.69	41.22 ± 0.53	<0.0001
PANSS total	98.05 ± 1.27	68.9 ± 0.92	<0.0001	79.65 ± 1.25	77.12 ± 1.27	0.18	72.63 ± 1.01	79.59 ± 1.40	0.00	55.01 ± 1.12	84.14 ± 0.99	<0.0001
PSP	44.98 ± 1.35	50.6 ± 1.05	0.001	44.90 ± 2.0	49.61 ± 0.94	0.03	47.56 ± 1.48	49.44 ± 1.04	0.47	59.24 ± 1.71	45.91 ± 0.90	<0.0001
CGI	5.98 ± 0.07	5.23 ± 0.06	<0.0001	5.52 ± 0.11	5.443 ± 0.06	0.69	5.63 ± 0.08	5.39 ± 0.07	0.05	4.472 ± 0.09	5.738 ± 0.05	<0.0001
CDSS	14.12 ± 1.55	12.82 ± 0.29	0.03	13.02 ± 0.61	13.25 ± 0.29	0.82	13.65. ± 0.49	13.03. ± 0.32	0.17	11.59 ± 0.39	13.67 ± 0.32	0.0003

Data show clinical scores (mean ± SEM) of the indicated patients before treatment as well as the statistical significance of the observed differences measured by the *p* value

*PANSS* Positive and Negative Syndrome Scale, *PPANSS* positive PANSS, *NPANSS* negative PANSS, *GPANSS* general psychopathology PANSS, *CDSS* Calgary Depression Scale for Schizophrenia, *PSP* Personal and Social Performance Scale, *CGI* Clinical Global Impression

In summary, applying a two-step hierarchical unsupervised classification method to FEP patients identified four patient subtypes characterized by different symptom profiles. C1A patients exhibited the most severe symptoms in all dimensions, and 57.70% of them were remitters after 4-week treatment with amisulpride, compared to 68.6% in the study sample as a whole (Table 1). In contrast, C2B patients exhibited less severe symptoms, and 90.4% of them were remitters after 4 weeks of treatment (Table 1).

### Validation of the clustering solution

The complexity of deriving clustering solutions makes validation crucial not only to ensure reproducibility but also to confirm that the derived clusters index clinically meaningful variations^43,44^. We first sought to validate our clustering solution using cross-validation, i.e., by first splitting data in a training and a test sample, and then assigning each patient of the test sample to one of the clusters derived from the training sample. Results from 50 independent random drawings showed that our clustering solution was robust with 86.8% to 95.5% of the patients (depending of the cluster) in the test sample being correctly classified (Supplementary Table 4).

As an alternative and complementary approach, we sought to validate our clustering solution on external biological measures, i.e., to investigate whether reducing clinical heterogeneity also reduces biological heterogeneity^45–47^. To this aim, we searched for serum biomarkers that were present at different levels between clusters. Univariate analysis did not identify serum proteins that distinguished C1B or C2A patients from the others. In contrast, C1A patients exhibited statistically higher levels of IL-7, IL-15, IL-17, IFN-γ, TNF-α, sICAM-1 and sVCAM-1 after correction for multiple test (Table 3). The probability that seven biomarkers or more would have been expressed at statistically higher levels in 97 randomly selected patients (to match the number of C1A patients) compared to the others was 5.47 × 10^−6^ as estimated by 10,000 successive random drawings (Supplementary Table 5). We also found that C2B patients exhibited lower levels of CXCL12 and higher levels of IL-8. Effect sizes were small to medium (0.5 > Cohen’s *d* coefficient > 0.2) for IFN-γ, IL-7, IL-17, TNF-α, sICAM-1, CXCL12 and sVCAM-1, and medium to high (1.0 > Cohen’s *d* coefficient > 0.5) for both IL-15 and IL-8. Because the *K-*sparse* clustering approach that we have used to define C1A, C1B, C2A and C2B subtypes was based on clinical features only, the fact that several peripheral biomarkers distinguished at least two patient subtypes from the others validated our clustering approach on external biological measures^48^.

**Table 3:** Differences in serum biomarker levels between patient subtypes

Biomarker	C1A versus others					C1B versus others					C2A versus others					C2B versus others				
	Mean		Univariate analysis			Mean		Univariate analysis			Mean		Univariate analysis			Mean		Univariate analysis		
	C1A	Others	Effect size	*P* value	FDR	C1B	Others	Effect size	*P* value	FDR	C2A	Others	Effect size	*P* value	FDR	C2B	Others	Effect size	*P* value	FDR
CCL2	2.39	2.42	−0.18	0.14	0.50	2.41	2.41	−0.04	0.77	0.93	2.43	2.41	0.14	0.25	0.85	2.42	2.41	0.08	0.53	0.84
CCL3	1.21	1.18	0.10	0.48	0.74	1.13	1.20	−0.35	0.04	0.46	1.20	1.19	0.05	0.69	0.90	1.21	1.18	0.09	0.47	0.82
CCL4	1.98	1.98	0.00	0.98	0.98	1.96	1.98	−0.09	0.51	0.87	1.98	1.97	0.06	0.65	0.90	1.98	1.98	0.01	0.93	0.95
CCL11	2.21	2.18	0.10	0.42	0.74	2.17	2.20	−0.13	0.39	0.86	2.20	2.19	0.06	0.63	0.90	2.18	2.19	−0.07	0.55	0.84
CCL13	2.10	2.09	0.03	0.82	0.87	2.06	2.10	−0.23	0.11	0.57	2.11	2.09	0.12	0.33	0.85	2.10	2.09	0.02	0.87	0.95
CCL17	2.43	2.47	−0.13	0.26	0.60	2.43	2.46	−0.10	0.50	0.87	2.47	2.45	0.05	0.67	0.90	2.50	2.44	0.19	0.16	0.46
CCL19	2.82	2.76	0.20	0.12	0.48	2.78	2.78	0.00	0.98	0.98	2.80	2.77	0.12	0.42	0.85	2.69	2.80	−0.29	0.01	0.12
CCL20	1.47	1.42	0.14	0.25	0.60	1.40	1.44	−0.16	0.31	0.79	1.41	1.44	−0.09	0.48	0.85	1.45	1.43	0.05	0.66	0.86
CCL22	3.08	3.06	0.12	0.31	0.60	3.04	3.07	−0.25	0.09	0.51	3.07	3.07	0.04	0.72	0.90	3.07	3.07	0.01	0.91	0.95
CCL26	0.80	0.81	−0.01	0.96	0.98	0.70	0.83	−0.29	0.04	0.46	0.84	0.79	0.11	0.38	0.85	0.85	0.79	0.14	0.29	0.67
CCL27	3.71	3.67	0.16	0.28	0.60	3.73	3.67	0.22	0.21	0.68	3.68	3.68	−0.01	0.94	0.97	3.60	3.71	−0.26	0.02	0.16
CX3CL1	4.29	4.27	0.06	0.72	0.86	4.32	4.27	0.16	0.37	0.86	4.31	4.27	0.14	0.37	0.85	4.19	4.30	−0.25	0.03	0.16
CXCL10	2.24	2.23	0.05	0.69	0.86	2.23	2.23	−0.01	0.96	0.98	2.24	2.23	0.02	0.88	0.97	2.22	2.24	−0.07	0.57	0.84
CXCL11	2.05	2.06	−0.05	0.68	0.86	2.03	2.07	−0.11	0.49	0.87	2.09	2.05	0.11	0.37	0.85	2.06	2.06	0.02	0.88	0.95
CXCL12	3.47	3.45	0.09	0.54	0.78	3.52	3.44	0.28	0.09	0.51	3.48	3.44	0.12	0.44	0.85	3.34	3.49	−0.36	1.54E−03	**2.69E−02**
IL-6	0.17	0.16	0.08	0.49	0.74	0.16	0.16	−0.07	0.62	0.87	0.16	0.16	0.00	0.97	0.97	0.16	0.16	−0.04	0.80	0.95
IL-7	1.22	1.15	0.36	3.53E−03	**2.47E−02**	1.16	1.17	−0.06	0.66	0.88	1.16	1.18	−0.11	0.37	0.85	1.14	1.18	−0.24	0.07	0.28
IL-8	1.01	1.07	−0.23	0.08	0.37	1.03	1.06	−0.08	0.61	0.87	1.00	1.08	−0.26	0.04	0.85	1.20	1.01	0.58	2.78E−06	**9.73E−05**
IL-10	0.13	0.12	0.14	0.23	0.60	0.13	0.12	0.02	0.85	0.96	0.11	0.13	−0.19	0.15	0.85	0.13	0.13	0.01	0.95	0.95
IL-12p40	1.93	1.90	0.13	0.29	0.60	1.90	1.91	−0.05	0.75	0.93	1.90	1.91	−0.02	0.85	0.97	1.89	1.91	−0.09	0.52	0.84
IL-15	0.51	0.45	0.50	8.01E-05	**2.81E−03**	0.45	0.48	−0.25	0.07	0.51	0.47	0.47	−0.04	0.72	0.90	0.44	0.48	−0.29	0.03	0.16
IL-16	2.30	2.31	−0.03	0.79	0.86	2.28	2.32	−0.16	0.26	0.71	2.31	2.31	0.03	0.82	0.97	2.33	2.30	0.14	0.27	0.67
IL-17	0.49	0.40	0.42	5.99E−04	**1.05E−02**	0.39	0.43	−0.20	0.15	0.60	0.39	0.44	−0.22	0.08	0.85	0.41	0.43	−0.07	0.60	0.84
IL-18	2.16	2.14	0.07	0.59	0.82	2.15	2.15	−0.01	0.96	0.98	2.16	2.15	0.05	0.72	0.90	2.12	2.16	−0.10	0.35	0.72
IL-21	0.99	0.99	0.03	0.78	0.86	0.98	0.99	−0.04	0.80	0.93	1.00	0.98	0.09	0.49	0.85	0.97	0.99	−0.10	0.41	0.76
IL-23	1.11	1.09	0.09	0.47	0.74	1.08	1.10	−0.09	0.52	0.87	1.11	1.09	0.11	0.39	0.85	1.07	1.10	−0.14	0.26	0.67
IL-27	3.03	3.02	0.04	0.74	0.86	3.02	3.02	0.01	0.94	0.98	3.05	3.01	0.12	0.36	0.85	2.97	3.03	−0.16	0.15	0.46
IFN-γ	0.81	0.70	0.40	1.08E−03	**1.26E−02**	0.69	0.74	−0.16	0.26	0.71	0.71	0.74	−0.09	0.48	0.85	0.68	0.74	−0.24	0.08	0.28
TNF-α	0.45	0.41	0.34	7.06E−03	**4.12E−02**	0.40	0.43	−0.21	0.15	0.60	0.42	0.42	−0.08	0.53	0.88	0.41	0.42	−0.11	0.37	0.72
TNF-β	0.08	0.07	0.13	0.36	0.65	0.07	0.08	−0.06	0.62	0.87	0.07	0.08	−0.13	0.34	0.85	0.08	0.08	0.06	0.63	0.85
VEGF	2.21	2.23	−0.06	0.61	0.82	2.23	2.22	0.05	0.75	0.93	2.22	2.22	0.01	0.93	0.97	2.23	2.22	0.02	0.87	0.95
sICAM-1	5.63	5.59	0.37	2.59E−03	**2.27E−02**	5.59	5.60	−0.07	0.58	0.87	5.59	5.60	−0.09	0.46	0.85	5.57	5.61	−0.26	0.05	0.22
sVCAM-1	5.83	5.80	0.30	1.45E−02	**7.26E−02**	5.80	5.81	−0.09	0.54	0.87	5.81	5.81	−0.01	0.92	0.97	5.79	5.82	−0.26	0.05	0.22
CRP	5.94	6.03	−0.16	0.22	0.60	6.19	5.96	0.38	0.00	0.17	5.95	6.02	−0.11	0.36	0.85	5.98	6.01	−0.04	0.77	0.95
SAA	6.30	6.40	−0.18	0.16	0.50	6.45	6.35	0.18	0.21	0.68	6.43	6.34	0.14	0.24	0.85	6.32	6.38	−0.12	0.37	0.72

Data show mean serum protein levels (log) in the indicated patient subtypes. Effect sizes, *p* values and FDRs are indicated for each comparison. FDR < 0.1 are given in bold values

*FDR* false discovery rate, *IL* interleukin, *TNF* tumor necrosis factor, *VEGF* vascular endothelial growth factor, *CRP* C reactive protein, *SAA* serum amyloid A protein, *sICAM-1* soluble intercellular adhesion molecule 1, *sVCAM-1* soluble vascular adhesion molecule 1

### Predicting remission in individual patient subtypes

As an attempt to identify serum biomarkers associated with remission in individual patient subtypes, we applied regularized logistic regression to clinical and biological data from C1A, C1B, C2A and C2B patients. None of the analyzed variables was associated with remission in C1B, C2A and C2B patients (not shown). In striking contrast, lower serum levels of IL-15, higher serum levels of CXCL12, seropositivity to CMV, use of recreational drugs and being younger were all associated with increased odds of being non-remitters in C1A patients (Table 4, model 1). Among these five variables, IL-15 was selected in 99.6% of the training/test runs and had a *p* value < 0.001. To estimate the predictive value of these five combined variables, we applied a regularized logistic regression to these five variables only (Table 4, model 2). All variables were selected more than 95% of the time and exhibited *p* values < 0.1. The predictive value of this model, assessed by the ROC curve was 73 ± 0.10%, and its specificity and selectivity were 45 ± 0.09% and 83 ± 0.03%, respectively.

**Table 4:** Clinical and biological variables associated with non-remission in C1A patients

Variable	Model 1			Model 2		
	Proportion of selection	Weighted mean odds ratio ± SD	*P* value	Proportion of selection	Weighted mean odds ratio ± SD	*P* value
Sex (being male)	0.315	1.156 ± 0.013	0.572			
Age	0.677	0.817 ± 0.008	**0.099**	0.953	0.804 ± 0.006	**0.095**
BMI	0.312	1.077 ± 0.004	0.654			
Waist circumference	0.341	1.068 ± 0.002	0.518			
Recreational drug use	0.774	1.240 ± 0.019	**0.070**	0.967	1.393 ± 0.016	**0.048**
Seropositivity to HSV-1	0.225	1.130 ± 0.012	0.757			
Seropositivity to CMV	0.702	1.283 ± 0.021	**0.093**	0.963	1.490 ± 0.028	**0.012**
Seropositivity to *Toxoplasma*	0.710	1.266 ± 0.025	0.119			
CCL2	0.168	1.029 ± 0.001	0.693			
CCL3	0.325	0.912 ± 0.004	0.690			
CCL4	0.162	1.012 ± 0.001	0.764			
CCL11	0.244	1.057 ± 0.003	0.643			
CCL13	0.148	1.036 ± 0.004	0.684			
CCL17	0.179	1.024 ± 0.009	0.517			
CCL19	0.359	0.976 ± 0.006	0.640			
CCL20	0.236	0.982 ± 0.001	0.763			
CCL22	0.151	0.971 ± 0.004	0.668			
CCL26	0.564	1.163 ± 0.010	0.291			
CCL27	0.154	1.048 ± 0.002	0.626			
CX3CL1	0.168	0.962 ± 0.002	0.784			
CXCL10	0.155	1.031 ± 0.004	0.754			
CXCL11	0.321	0.937 ± 0.002	0.634			
CXCL12	0.715	1.240 ± 0.019	**0.077**	0.964	1.369 ± 0.012	**0.020**
IL-6	0.416	0.913 ± 0.002	0.656			
IL-7	0.183	0.950 ± 0.002	0.803			
IL-8	0.483	1.148 ± 0.010	0.528			
IL-10	0.372	1.093 ± 0.004	0.743			
IL-12p40	0.281	0.947 ± 0.002	0.591			
IL-13	0.284	0.953 ± 0.001	0.717			
IL-15	0.996	0.756 ± 0.009	**5.00E−04**	1.000	0.585 ± 0.005	**4.00E−04**
IL-16	0.288	0.933 ± 0.002	0.711			
IL-17	0.405	0.941 ± 0.002	0.520			
IL-18	0.18	1.000 ± 0.003	0.640			
IL-21	0.211	0.962 ± 0.002	0.812			
IL-23	0.256	0.957 ± 0.002	0.709			
IL-27	0.238	1.067 ± 0.004	0.796			
IFN-γ	0.21	0.954 ± 0.002	0.689			
TNF-α	0.553	1.226 ± 0.016	0.240			
TNF-β	0.32	0.946 ± 0.001	0.664			
VEGF	0.134	1.025 ± 0.002	0.596			
sICAM-1	0.266	0.924 ± 0.003	0.669			
sVCAM-1	0.474	0.897 ± 0.003	0.388			
CRP	0.67	0.841 ± 0.005	0.186			
SAA	0.088	0.973 ± 0.000	0.233			

Data show the variables that were included in regularized regression logistic models. All variables listed in the left column were included in model 1. Recreational drug use, seropositivity to CMV, IL-15, CXCL12 and age were included in model 2. For each variable, the proportion of drawings (out of 2000) in which the variable was selected is indicated, as well as the weighted mean odds ratio (ORs) ± SD. The *p* values were computed adjusting a single elastic net model with the mean *α* and mean *λ* as hyper-parameters. In model 1, mean *α* and mean *λ* hyper-parameters were 0.37 ± 0.29 and 0.26 ± 0.28 respectively. Mean area under the curve (AUC) was 0.62 ± 0.09 with minimal and maximal values of 0.42 and 0.82 respectively. In Model 2, mean *α* and mean *λ* hyper-parameters were 0.11 ± 0.07 and 0.13 ± 0.13 respectively. Mean AUC was 0.73 ± 0.1 with minimal and maximal values of 0.36 and 0.95 respectively. The *p* values < 0.1 are given in bold values

*BMI* bone marrow index, *CMV* cytomegalovirus, *HSV-1* herpes simplex virus type 1, *IL* interleukin, *TNF* tumor necrosis factor, *VEGF* vascular endothelial growth factor, *CRP* C reactive protein, *SAA* serum amyloid A protein, *sICAM-1* soluble intercellular adhesion molecule 1, *sVCAM-1* soluble vascular adhesion molecule 1

## Discussion

Heterogeneity of patients with mental disorders may impede identification of adequate predictors of remission^43^. In keeping with this hypothesis, we have failed to identify serum biomarkers associated with remission in non-stratified FEP patients. To overcome this problem, we used a hierarchical clustering approach to identify subtypes of patients based on their clinical symptoms. Several unsupervised clustering methods have been used to stratify patients with mental disorders based on clinical symptoms and case history variables^49–53^ or social cognitive measures^54^. Given a set of data points, clustering methods aim to partition data into a specified number (*k*) of clusters, such that the samples in each cluster are more similar to one another than to those in the other clusters. This entails defining a measure of similarity or distance between data points. As recently pointed out^43^, the outcome of clustering is highly dependent on the input data with relatively little convergence towards a coherent and consistent set of subtypes. Unfortunately, the biological relevance of the few subtypes identified so far was generally limited and did not clearly reflect underlying biological mechanisms. Here, we have used a two-step hierarchical unsupervised clustering method to stratify FEP patients into four subtypes, termed C1A, C1B, C2A and C2B, based on their clinical symptoms. C1A patients were characterized by the most severe symptoms in the positive, negative and general physiopathology dimensions. In contrast, C2B patients were the least severely affected. Most importantly, C1A and C2B patients did not only differ from other patients in terms of symptoms severity but also exhibited specific peripheral immune signatures suggesting that these subtypes reflected distinct pathophysiological entities^45,55^. Our study therefore provides the proof of concept that clustering methods aimed at reducing clinical heterogeneity may also reduce biological heterogeneity.

Several authors in the field have tried to stratify psychosis spectrum patients on the basis of symptoms. In a pioneer study, Dollfus et al.^51^ have used the Ward’s method of hierarchical clustering to identify four subtypes of schizophrenia patients that they called “positive”, “negative”, “mixed” and “disorganized”. These four subtypes are very similar to the four subsets that we describe here, with our C1B, C2A, C1A and C2B subsets being very similar to Dollfus’ “positive”, “negative”, “mixed” and “disorganized” subtypes, respectively.

In contrast to the current ”one size fits all” or ”trial and error” approach in healthcare, stratified medicine aims at sorting a population into biologically relevant subtypes. We found that the vast majority (90.4%) of C2B patients were remitters after treatment. This agrees with previous studies which have shown that patients with less severe negative symptoms are more likely to be remitters than others^9^. In contrast, the proportion of remitters among C1A, C1B and C2B patients ranged between 54.8% and 61.3%. Thus, the clustering solution that we describe here constitutes a first step towards stratified medicine for psychotic patients.

In support of a critical role of inflammation in psychiatric diseases, add-on treatments with anti-inflammatory drugs have been tested in severe and treatment-resistant psychiatric patients^56–64^. For example, acetyl salicylic acid (Aspirin) which interrupts the immuno-inflammatory cascade by inhibiting cyclooxygenase (COX)-1 and COX-2 showed promising results as an add-on treatment of schizophrenia in comparison to treatment as usual^60,63^. In most cases however, add-on anti-inflammatory treatments in psychotic patients only provided modest improvements in clinical outcome. This could be explained if only a subtype of the treated patients exhibited a pro-inflammatory profile at baseline. In agreement with this latter hypothesis, an add-on trial in patients with psychotic disorders showed that those with increased CRP levels had the largest response to add-on Aspirin as compared to those with lower levels^65^. Compared to others, C1A patients exhibited higher levels of IL-7, IL-15, IL-17, IFN-γ, TNF-α, sICAM-1 and sVCAM-1. Therefore, a reasonable and testable hypothesis is that C1A patients would be those that could benefit the most from add-on anti-inflammatory treatment. On another topic, several authors have proposed that inflammation was associated with poor clinical outcome in psychosis^66–69^. In agreement with these studies, C1A patients were both characterized by higher levels of several inflammatory biomarkers and a lower proportion of non-remitters (57.7% compared to 68.6% in the study sample as a whole, and 90.4 % in the C2B subtype).

Compared to stratified medicine, personalized medicine builds on a finer sub-classification of patients to enable individual tailoring of treatment to maximize response. Bearing this in mind, we have used regularized logistic regression to select variables that could predict remission in individual patient subtypes. In C1A patients but not in others, lower levels of IL-15, higher levels of CXCL12, recreational drug use, being seropositive to CMV and being younger were all associated with increased odds of being non-remitters after adjustment for covariates suspected to impact cytokine levels or response to treatment. While IL-15 is mainly known for its role in regulating natural killer and T cells^70,71^, it is also produced by astrocytes and neural progenitors^72,73^, regulates neurogenesis and exerts anti-depressive effects in mice^74,75^. Likewise, while CXCL12 was first described as a chemotactic factor for lymphocytes and macrophages, it is also secreted by glial cells and neurons and plays a role in brain plasticity and function^76^. One of the two CXCL12 receptors, CXCR4, acts at both the synaptic and post-synaptic levels by promoting the release of glutamate and γ-aminobutyric acid (GABA) and by activating the voltage-gated K channel Kv2.1, respectively. Whether and how IL-15 and CXCL12 impact response to antipsychotics remains to be elucidated.

In addition to lower levels of IL-15 and higher levels of CXCL12, being seropositive to CMV and the use of recreational drugs were both associated with an increased risk of being non-remitters in C1A patients. Previous studies have identified CMV infection^77^ and use of recreational drugs^78^ as risk factors for schizophrenia. Why these two variables are also associated with an increased risk of being non-remitters in C1A patients is unclear.

Our results, if replicated, could pave the way for the development of a blood-based assisted clinical decision support system for selecting the most appropriate treatment in psychotic patients.

## Supplementary information

Legends to Supplementary Figures and Tables

Supplementary Table 1

Supplementary Table 2

Supplementary Table 3

Supplementary Table 4

Supplementary Table 5

Supplementary Figure 1
