# Correction: Stratification and prediction of remission in first-episode psychosis patients: the OPTiMiSE cohort study

**DOI:** 10.1038/s41398-019-0505-7

**Published:** 2019-06-21

**Authors:** Emanuela Martinuzzi, Susana Barbosa, Douglas Daoudlarian, Wafa Bel Haj Ali, Cyprien Gilet, Lionel Fillatre, Olfa Khalfallah, Réjane Troudet, Stéphane Jamain, Guillaume Fond, Iris Sommer, Stefan Leucht, Paola Dazzan, Philip McGuire, Celso Arango, Covadonga M. Diaz-Caneja, Wolfgang Fleischhacker, Dan Rujescu, Birte Glenthøj, Inge Winter, René Sylvain Kahn, Robert Yolken, Shon Lewis, Richard Drake, Laetitia Davidovic, Marion Leboyer, Nicolas Glaichenhaus, the OPTiMiSE Study Group

**Affiliations:** 1 Université Côte d’Azur, Centre National de la Recherche Scientifique, Institut de Pharmacologie Moléculaire et Cellulaire, Valbonne, France; 2 Université Paris Est Créteil, Faculté de Medicine Institut, National de la Santé et de la Recherche Médicale, Créteil, France; 3 Université Côte d’Azur, Centre National de la Recherche Scientifique, Laboratoire Informatique Signaux et Systèmes de Sophia Antipolis, Sophia Antipolis, France; 4 Assistance Publique Hôpitaux de Marseille, Marseille, France; 5 Department of Neuroscience and Department of Psychiatry, University Medical Center Groningen, Rijks Universiteit Groningen, Groningen, The Netherlands; 6 Department of Medical and Biological Psychology, University of Bergen, Bergen, Norway; 7 Department of Psychiatry and Psychotherapy, Technische Universität München, München, Germany; 8 Department of Psychosis Studies, Institute of Psychiatry, National Institute for Health Research, Mental Health Biomedical Research Centre, King’s College London, London, UK; 9 Child and Adolescent Psychiatry Department, Hospital General Universitario Gregorio Marañón, Universidad Complutense, Madrid, Spain; 10 Department of Psychiatry, Psychotherapy and Psychosomatic Medicine, Medical University Innsbruck, Innsbruck, Austria; 11 Department of Psychiatry, University of Halle, Halle, Germany; 12 Faculty of Health and Medical Sciences, Center for Neuropsychiatric Schizophrenia Research and Center for Clinical Intervention and Neuropsychiatric Schizophrenia Research, Psychiatric Hospital Center Glostrup, University of Copenhagen, Copenhagen, Denmark; 13 Department of Psychiatry, Brain Center Rudolf Magnus, UMC Utrecht, Utrecht, The Netherlands; 14 John Hopkins School of Medicine, The John Hopkins Hospital, Baltimore, USA; 15 Division of Psychology and Mental Health, School of Health Sciences, Faculty of Biology, Medicine and Health, Manchester Academic. Health Sciences Centre (MAHSC), University of Manchester, Manchester, UK; 16 Assistance Publique Hôpitaux de Paris, Pole de Psychiatrie et Addictologie, Hopitaux Universitaires Henri Mondor, Créteil, France; 17 Fondation Fondamental, Hôpital Albert Chenevier Pôle de Psychiatrie, Créteil, France

**Keywords:** Biomarkers, Physiology

**Correction to: Translational Psychiatry**

10.1038/s41398-018-0366-5 Published online 17 January 2019

The original Article did not feature the list of collaborators. This has now been corrected in the PDF and HTML versions of this Article.

