# Supplementary material for: Stratification and prediction of remission in first-episode psychosis patients: the OPTiMiSE cohort study
Source: Transl Psychiatry. 2019 Jan 17;9:20. doi: 10.1038/s41398-018-0366-5 (PMC6336802; doi:10.1038/s41398-018-0366-5)
Supplement: Supplementary file 1 — Legends to Supplementary Figures and Tables [file 41398_2018_366_MOESM1_ESM.pdf]

Legends to Supplementary Figures and Tables

Supplementary Figure 1: *K*-sparse* clustering method.

To stratify m patients into k clusters based on their PANSS scores (d items per patient), we prepared a matrix X with one patient per line and one PANSS item per column. Our objective was to find a matrix Y of labels. We thus tried to solve an optimization problem for finding a space which discriminated clusters based on a limited number of weighted PANSS items. The output was a W (weight) matrix with k columns and d lines computing the weight of each PANSS item. We achieved this goal by using an alternating minimization procedure on Y and W in which we tried to minimize the Frobenius norm [38].

Supplementary Table 1: Serum protein concentration descriptive statistics. The LLOD for the indicated protein, the proportion of serum samples (out of 325) in which protein levels were < LLOD, the minimum, maximum, median and mean values, and standard error of the mean (SEM) are indicated.

Supplementary Table 2: Univariate analysis of serum protein levels in non-remitters and remitters. For each protein, mean serum levels (Log) in non-remitters and remitters are indicated. Effect sizes and FDRs are also shown.

Supplementary Table 3: PANSS items selected by *K*-sparse*. The 30 items of the PANSS scale are indicated. The weight of items selected by *K*-sparse* to discriminate C1 and C2 patients (1st level clustering), C1A versus C1B patients, and C2A versus C2B patients (2nd level clustering) are indicated.

Supplementary Table 4: Cross-validation of the K-sparse* clustering solution. We randomly split the 325 patients of the study sample in a training and a test set of 243 (75%) and 82 (25%) patients respectively. We performed a two-step hierarchical clustering of patients from the training set and identified four clusters, C1A', C1B', C2A' and C2B'. We classified each patient of the test set in one of the four clusters based on the distance to each cluster centroid. We then computed for each patient of the test set originally classified in the C1A, C1B, C2A or C2B cluster, the proportion who were classified in C1A', C1B', C2A' and C2B'. For example, among patients of the test set who belonged to the C1A cluster, 95.49% were correctly classified in the C1A' cluster, and 3.46% and 1.05% were incorrectly classified in the C1B' and C2A' cluster respectively. Data show the results of 50 random drawings.

Supplementary Table 5: Probability that C1A patients would express statistically higher levels of serum biomarkers compared to others. We performed a simulation by drawing 10,000 times a random selection of 97 patients (to match the number of patients in the C1A subtype) out of 325. We then computed the number of serum proteins that passed the FDR test when comparing the selected patients versus the others. The Table shows the probability that n serum proteins are expressed at higher levels in C1A patients compared to others with n values ranging from 0 to 12.
