# Supplementary material for: Stratification and prediction of remission in first-episode psychosis patients: the OPTiMiSE cohort study
Source: Transl Psychiatry. 2019 Jan 17;9:20. doi: 10.1038/s41398-018-0366-5 (PMC6336802; doi:10.1038/s41398-018-0366-5)
Supplement: Supplementary file 3 — Supplementary Table 2 [file 41398_2018_366_MOESM3_ESM.pdf]

Supplementary Table 2

	mean levels (Log)		univariate analysis	
Biomarker	Non-remitters	Remitters	effect size	FDR
CCL2	2.43	2.41	-0.15	0.16
CCL3	1.16	1.20	0.16	0.44
CCL4	1.97	1.98	0.05	0.62
CCL11	2.19	2.19	0.03	0.83
CCL13	2.09	2.10	0.06	0.42
CCL17	2.43	2.47	0.15	0.38
CCL19	2.79	2.77	-0.05	0.65
CCL20	1.43	1.44	0.01	0.96
CCL22	3.06	3.07	0.09	0.48
CCL26	0.82	0.80	-0.04	0.77
CCL27	3.69	3.68	-0.05	0.45
CX3CL1	4.30	4.27	-0.13	0.82
CXCL10	2.24	2.23	-0.05	0.65
CXCL11	2.05	2.07	0.06	0.25
CXCL12	3.49	3.43	-0.19	0.22
IFN-γ	0.73	0.73	-0.02	0.97
IL-6	0.16	0.16	0.05	0.55
IL-7	1.19	1.16	-0.15	0.25
IL-8	1.04	1.06	0.05	0.66
IL-10	0.13	0.12	-0.06	0.61
IL-12p40	1.89	1.91	0.07	0.51
IL-15	0.47	0.47	-0.02	0.88
IL-16	2.28	2.32	0.20	0.15
IL-17	0.42	0.43	0.01	0.96
IL-18	2.16	2.15	-0.04	0.84
IL-21	0.99	0.99	0.00	0.91
IL-23	1.08	1.10	0.13	0.47
IL-27	3.02	3.02	-0.02	0.71
sICAM-1	5.59	5.60	0.08	0.64
sVCAM-1	5.81	5.81	0.04	0.89
TNF-α	0.44	0.42	-0.18	0.14
TNF-β	0.08	0.08	-0.10	0.48
VEGF	2.24	2.21	-0.11	0.41
SAA	6.35	6.37	0.04	0.92
CRP	5.95	6.02	0.13	0.21
