# Supplementary material for: Stratification and prediction of remission in first-episode psychosis patients: the OPTiMiSE cohort study
Source: Transl Psychiatry. 2019 Jan 17;9:20. doi: 10.1038/s41398-018-0366-5 (PMC6336802; doi:10.1038/s41398-018-0366-5)
Supplement: Supplementary file 4 — Supplementary Table 3 [file 41398_2018_366_MOESM4_ESM.pdf]

Supplementary Table 3

PANSS item	Dimension	C1 versus C2	C1A versus C1B	C2A versus C2B
P1	Delusions		2.05	1.96
P2	Conceptual disorganisation			
P3	Hallucinatory behaviour		3.60	1.96
P4	Excitement		0.73	
P5	Grandiosity			
P6	Suspiciousness		1.58	2.85
P7	Hostility			
N1	Blunted affect	0.92		
N2	Emotional withdrawal	3.92		
N3	Poor rapport	0.79		
N4	Passive social withdrawal	5.52		
N5	Difficulty in abstract thinking			
N6	Lack of spontaneity	0.54		
N7	Stereotyped thinking			
G1	Somatic concern			
G2	Anxiety		1.97	1.91
G3	Guilt feelings			
G4	Tension			
G5	Mannerism and posturing			
G6	Depression			
G7	Motor retardation			
G8	Uncooperativeness			
G9	Unsual thought content		2.37	1.64
G10	Disorientation			
G11	Poor attention	0.86		
G12	Lack of judgement and insight			
G13	Disturbance of volition	1.14		
G14	Poor impulse control			0.63
G15	Preoccupation	4.66	1.12	
G16	Active social avoidance	1.43	0.82	1.30
