# Supplementary material for: Stratification and prediction of remission in first-episode psychosis patients: the OPTiMiSE cohort study
Source: Transl Psychiatry. 2019 Jan 17;9:20. doi: 10.1038/s41398-018-0366-5 (PMC6336802; doi:10.1038/s41398-018-0366-5)
Supplement: Supplementary file 5 — Supplementary Table 4 [file 41398_2018_366_MOESM5_ESM.pdf]

Supplementary Table 4

Confusion matrix		Cluster to which patients from the testing set are classified			
		C1A'	C1B'	C2A'	C2B'
Cluster to which patients from the testing set belong to	C1A	**0.9549**	0.0346	0.0105	0.0000
	C1B	0.1012	**0.8872**	0.0086	0.0030
	C2A	0.0167	0.0113	**0.8681**	0.1039
	C2B	0.0000	0.0021	0.0676	**0.9303**
