# Supplementary material for: Stratification and prediction of remission in first-episode psychosis patients: the OPTiMiSE cohort study
Source: Transl Psychiatry. 2019 Jan 17;9:20. doi: 10.1038/s41398-018-0366-5 (PMC6336802; doi:10.1038/s41398-018-0366-5)
Supplement: Supplementary file 6 — Supplementary Table 5 [file 41398_2018_366_MOESM6_ESM.pdf]

**Supplementary Table 5**

| Nb. of proteins expressed at higher levels in C1A patients | Probability |
|---|---|
| 0 | 9.16E-01 |
| 1 | 2.88E-02 |
| 2 | 3.93E-03 |
| 3 | 7.25E-04 |
| 4 | 1.31E-04 |
| 5 | 5.00E-05 |
| 6 | 7.81E-06 |
| 7 | 5.47E-06 |
| 8 | 0.00E+00 |
| 9 | 1.95E-07 |
| 10 | 0.00E+00 |
| 11 | 0.00E+00 |
| 12 | 2.44E-08 |
