# Supplementary material for: Stratification and prediction of remission in first-episode psychosis patients: the OPTiMiSE cohort study
Source: Transl Psychiatry. 2019 Jan 17;9:20. doi: 10.1038/s41398-018-0366-5 (PMC6336802; doi:10.1038/s41398-018-0366-5)
Supplement: Supplementary file 7 — Supplementary Figure 1 [file 41398_2018_366_MOESM7_ESM.pdf]

Supplementary Figure 1

Problem statement

— Let define m the number of patients, d the number of features and k the number of clusters.

— Let define $Y \in \{0,1\}^{m \times k}$ the indicator matrix such that

$$Y_{ij} = \begin{cases} 1 & \text{if patient } i \text{ belongs to cluster } j \\ 0 & \text{otherwise} \end{cases}$$

— Let define $X \in \mathbb{R}^{m \times d}$ the matrix containing the PANSS scores of each patient.

— Let define $W \in \mathbb{R}^{d \times k}$ a projection matrix which aims to select relevant features.

K-sparse* optimization problem

$$\min_{W,Y} \ \frac{1}{2}\|Y - XW\|_F^2 \quad \text{s.t} \quad \|W\|_1 \leq \eta \tag{1}$$

Alternating minimization : We consider the optimization problem (1) and we perform an alternating minimization :

1. Y labels are fixed, we compute a sparse W matrix in order to select relevant features. The feature $j \in \{1, \ldots, d\}$ is selected if $\|W(j,:)\| > 0$.
2. W is fixed, we compute new labels Y in the new space XW.

Algorithm 1 K-sparse* algorithm.

Input : $X, Y, L, N, k, \gamma, \eta$
for $l = 0, \ldots, L$ **do**
  for $n = 0, \ldots, N$ **do**
    $V = W - \gamma X^T(XW - Y)$
    $W \leftarrow P_\eta^1(V)$
  end for
  $Y \leftarrow \texttt{kmeans}(XW, k)$
end for
Output : Y, W
